# Supplementary material for: Modalities Differentiation of Pain Perception Following Ischemic Stroke: Decreased Pressure Pain Perception
Source: Biomedicines. 2025 Sep 11;13(9):2241. doi: 10.3390/biomedicines13092241 (PMC12466989; doi:10.3390/biomedicines13092241)
Supplement: Supplementary file 1 [file biomedicines-13-02241-s001.zip › biomedicines-3708338-supplementary.pdf]

Supplementary Materials

Table S1. Detailed clinical characteristics of stroke patients

Patient	Age	Sex	Time past stroke onset (day)	Stroke site	MRS	NIHSS	Disease History
1	70	male	18	thalamus	0	1	Diabetes
2	60	female	24	thalamus	0	1	Fatty liver
3	27	male	28	basal ganglia	0	1	Hypertension
4	55	male	16	cerebellum	0	1	–
5	46	male	32	thalamus	/	/	/
6	55	male	30	basal ganglia	0	1	Hypertension
7	38	male	32	basal ganglia	0	3	–
8	77	male	11	frontal lobe	0	0	Hypertension
9	43	male	18	frontal lobe	/	0	–
10	46	male	20	frontal lobe	1	0	–
11	37	male	6	TPJ	0	3	Diabetes
12	58	male	10	thalamus	1	0	–
13	71	male	12	frontal lobe	1	0	Hypertension
14	65	male	12	right LI	1	1	Hypertension
15	47	male	9	cerebellum	0	1	–
16	68	male	7	basal ganglia	2	5	–
17	48	male	7	left LI	0	1	Diabetes
18	53	male	3	frontal lobe	1	1	Coronary disease
19	60	male	9	PLIC	0	6	Hypertension
20	54	male	8	basal ganglia	1	1	Hypertension
21	55	male	5	PLIC	0	2	–
22	54	male	3	frontal lobe	0	0	Hypertension
23	60	male	5	thalamus	0	1	Diabetes
24	56	male	4	thalamus	0	0	Hypertension
25	45	female	27	cerebellum	0	1	–
26	59	male	5	thalamus	0	2	Hypertension
27	71	male	3	basal ganglia	0	1	–
28	54	male	7	PLIC	0	3	Hypertension
29	52	male	6	pons	0	0	–
30	72	male	12	basal ganglia	0	1	–

Note: *TPJ = temporoparietal junction; LI = lacunar infarction; PLIC = posterior limb of the internal capsule*

/ means not measured; – means none
